# Identification of *qnrVF* in a Multidrug-Resistant Vibrio furnissii Clinical Strain

**DOI:** 10.1128/spectrum.01934-22

**Published:** 2023-01-19

**Authors:** Yao Wang, Weidong Li, Qingyan Deng, Yueming Huang, Xinhao Zhou, Zhifen Guan, Zhifeng Yang, Lijun Xiang, Yanhong Chen

**Affiliations:** a Department of Gastrointestinal Surgery, Zhongshan People’s Hospital, Zhongshan, People’s Republic of China; b Department of Hospital Infection Control, Zhongshan People’s Hospital, Zhongshan, People’s Republic of China; Health Canada

**Keywords:** *qnrVF*, IS*CR1*, *Vibrio*

## Abstract

We found a new *qnr* gene, *qnrVF1*, carried by a multidrug resistance plasmid in a clinical Vibrio furnissii isolate. QnrVF1 exhibits 44.6% to 72.5% similarity in identity with other Qnr family proteins. *QnrVF* alleles are mainly encoded by chromosomes of V. furnissii and Vibrio fluvialis. Phylogenic analysis showed that QnrVF1 and QnrVF2 form a distinct clade in Qnr proteins. Thus, *qnrVF* represents a new *qnr* family. In addition, the *qnrVF1* gene is often flanked by the mobile element IS*CR1*. Thus, it is likely that *qnrVF1* is mobilized by IS*CR1* from chromosome to plasmid in V. furnissii.

**IMPORTANCE** Quinolones are widely used drugs. Bacteria contain a quinolone resistance gene, which mediates resistance to quinolones. Currently, seven families of Qnr proteins, QnrVC, QnrA, QnrB, QnrC, QnrD, QnrE, and QnrS, have been identified. However, it is unclear whether there are any other *qnr* families. In this study, we identified a new *qnr* family, *qnrVF*. We found many V. furnissii and V. fluvialis strains that possess chromosomal *qnrVF* alleles, suggesting that V. furnissii and V. fluvialis are the reservoirs of *qnrVF*. We also found that QnrVF1 confers low-level resistance to quinolones. IS*CR1* may facilitate the spread of *qnrVF1*. The emergence and spread of *qnrVF* may pose a considerable threat to public health.

## OBSERVATION

Qnr is a pentapeptide repeat protein that confers resistance to quinolones. Currently, there are seven families of Qnr proteins, QnrVC, QnrA, QnrB, QnrC, QnrD, QnrE, and QnrS (1). Studies have shown that many *Vibrio* strains carry quinolone resistance genes, *qnrVC* alleles, and that they possess mutations in the quinolone resistance-determining regions *gyrA* and *parC*. These quinolone resistance genes and mutations in *gyrA* and *parC* produce fluoroquinolone resistance in *Vibrio* species (1, 2). Vibrio furnissii is phylogenetically close to Vibrio fluvialis and Vibrio cholerae (3). V. furnissii can cause acute gastroenteritis, bacteremia, cellulitis, and skin lesions (4–6). In this study, we identify a new family, QnrVF, of Qnr proteins. QnrVF alleles are mainly encoded by chromosomes of V. furnissii and V. fluvialis. The *qnrVF1* gene is often flanked by the mobile element IS*CR1*. It is likely that *qnrVF1* is mobilized by IS*CR1* from the chromosome to the plasmid of V. furnissii. Emergence of this mobile quinolone resistance gene constitutes a threat to public health.

In August 2018, strain 104486766 was obtained from a stool sample of a 63-year-old male patient in Zhongshan People’s Hospital, China. Strain 104486766 was identified as V. furnissii by 16S rRNA gene sequencing and API20E. Antimicrobial susceptibility testing was conducted using a broth dilution method based on the CLSI guidelines (7). The results of antimicrobial susceptibility testing for V. furnissii 104486766 showed this strain to be resistant to azithromycin, ampicillin, ciprofloxacin, tetracycline, and cephalosporins, including ceftiofur, ceftriaxone, and ceftazidime. Aztreonam, chloramphenicol, meropenem, imipenem, streptomycin, and tigecycline were found to effectively inhibit the growth of strain 104486766 (see Table S1 in the supplemental material). To determine whether the cephalosporins and azithromycin resistance phenotypes are transferable, a conjugation assay was performed, as described in a previous work (8). Specifically, 100 μL of strain 104486766 and 1,000 μL of Escherichia coli J53 (sodium azide resistance) overnight cultures were mixed together. The pellet was obtained by centrifugation and then resuspended in 70 μL Luria-Bertani (LB) broth and spotted on a filter membrane. The filter membrane was put on the LB agar plate and incubated at 37°C for 12 to 18 h. Next, the cell mixture was washed from the filter membrane, and 100 μL culture was spread on eosin-methylene blue agar plates containing 200 mg/L sodium azide plus 4 mg/L ceftazidime (or 100 mg/L azithromycin). However, after three independent experiments, no transconjugant was obtained.

Given that only a few clinical V. fluvialis strains, isolated from India, have been reported to be able to withstand azithromycin, ciprofloxacin, and cephalosporins simultaneously (9), the ability of V. furnissii 104486766 to confer coresistance to these drugs drew our attention. To understand the mechanisms underlying the resistance shown by V. furnissii 104486766, we sequenced its genome and performed bioinformatic analysis. Briefly, the genomic DNA of V. furnissii 104486766 was sequenced using HiSeq 2000 and Oxford Nanopore MinION systems. Using Unicycler, short-read and long-read data were assembled using a hybrid assembly strategy, and then, the complete genome sequences of V. furnissii 104486766 were obtained (10). BLAST Ring Image Generator (11), RAST (https://rast.nmpdr.org), ResFinder, PlasmidFinder, and Easyfig (12) were used to compare sequences, annotate the bacterial genome, identify antibiotic resistance genes, detect plasmid replicons, and generate images of comparative sequences, respectively.

**FIG 1:**
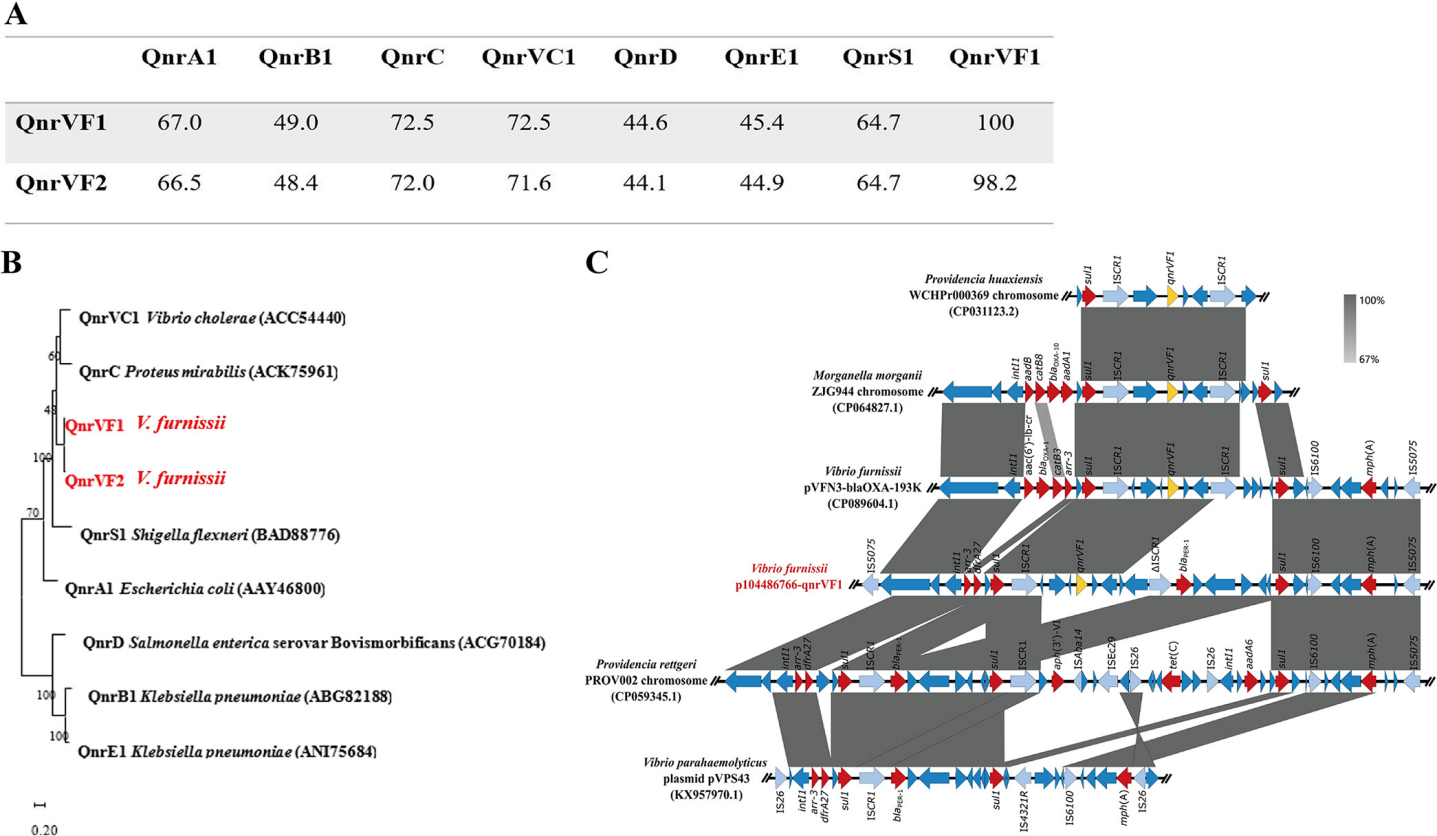
*QnrVF1* is likely mobilized by IS*CR1* from the chromosome to the plasmid of V. furnissii. (A) Sequence identities (%) of Qnr proteins. (B) Phylogenetic tree of Qnr proteins. The phylogenetic tree was constructed using the maximum-likelihood method with MEGA X. Numbers at nodes are percentage bootstrap values based on 500 replications. Bar, 0.2 difference at the amino acid level. (C) Linear comparisons of the *qnrVF1*, *mph*(A), and *bla*_PER-1_ genetic environment in p104486766-qnrVF1 with other related sequences available in the NCBI database. Yellow arrows indicate the *qnrVF1* gene, blue arrows indicate mobile elements and nondrug resistance genes, and red arrows indicate other drug resistance genes.

We found that V. furnissii 104486766 harbors a 198,507-bp plasmid, p104486766-qnrVF1, and two chromosomes. Here, two versions of the *Qnr*-like gene, *qnrVF1* and *qnrVF2*, were detected in p104486766-qnrVF1 and chromosome II, respectively. QnrVF1 and QnrVF2 were quite similar, sharing 98.2% identity. However, QnrVF1 showed 44.6% to 72.5% similarity in identity with other reported Qnr family proteins, and the similarity in the identity of QnrVF2 with other Qnr family proteins ranged from 44.1% to 71.6% (Fig. 1A). Phylogenetic analysis showed that QnrVF1 and QnrVF2 form a distinct branch among Qnr proteins (Fig. 1B). All of this indicates that QnrVF represents a new family of Qnr proteins. BLAST analysis, conducted in March 2022, showed that only V. furnissii 104486766 and V. furnissii VFN3 carried plasmid-borne *qnrVF1*, while three Morganella morganii strains and Providencia huaxiensis WCHPr000369 harbored chromosomal *qnrVF1*. In contrast, the other 20 V. furnissii strains, more than 60 V. fluvialis strains, and 5 V. cholerae strains harbored chromosomal *qnrVF* alleles. The levels of identity of QnrVF1 and chromosomal QnrVF alleles ranged from 96.8% to 99.1%. In p104486766-qnrVF1, pVFN3-blaOXA-193K (hosted by V. furnissii VFN3), Providencia huaxiensis WCHPr000369, and Morganella morganii ZJG944, *qnrVF1* genes were all flanked by IS*CR1* (Fig. 1C). These results suggest that V. furnissii and V. fluvialis may be the reservoirs of *qnrVF*, and *qnrVF1* is mobilized by IS*CR1* from the chromosome to p104486766-qnrVF1 and pVFN3-blaOXA-193K of V. furnissii. To determine whether *qnrVF1* is functional, we designed primers (F-CGGGATCCatgaaattaaatcatcacac and R-CGAGCTCttagtcaggaaccacaatc) and cloned *qnrVF1* into pET28a. Results from MIC studies showed that, compared with negative control, ciprofloxacin and nalidixic acid resistance levels were 4- and 8- fold higher, respectively, when E. coli BL21(DE3) expressed QnrVF1 (Table S2).

p104486766-qnrVF1 is an IncA/C2 replicon type plasmid. It encodes 11 antibiotic resistance genes, including *aac(3′)IId*, *floR*, *mph*(A), *sul1*, *sul2*, *tet*(D), *qnrVF1*, and *bla*_PER-1_ (Fig. S1). These antibiotic resistance genes found in p104486766-qnrVF1 probably mediate V. furnissii 104486766’s resistance to many antibiotics (Table S1). BLAST analysis revealed that p104486766-qnrVF1 shares 100% identity with pKP-16-57-NDM-1, pEc-13-49-NDM-1, pVFN3-blaOXA-193K, pPV835TEM24, and pVC1447, while coverages were found to range from ≈65% to 96%. Among these plasmids, only p104486766-qnrVF1 and pVFN3-blaOXA-193K harbored *qnrVF1.* p104486766-qnrVF1 and pVFN3-blaOXA-193K harbored different antibiotic resistance genes (Fig. 1C; Fig. S1). The genetic structure of *mph*(A) and *bla*_PER-1_ in p104486766-qnrVF1 was found to differ from those of pVPS43 (hosted by V. parahaemolyticus) and the chromosomal region of Providencia rettgeri PROV002 (Fig. 1C). *IntI1*, *tnpA*, IS*CR1*, and IS*5075* surrounded *mph*(A) and *bla*_PER-1_ in p104486766-qnrVF1. These mobile elements may facilitate the spread of these antibiotic resistance genes.

Previous studies showed that aquatic bacteria are the source of *qnrA*, *qnrC*, *qnrS*, and *qnrVC* (1, 13). In agreement with these findings, two water-dwelling bacteria, V. furnissii and V. fluvialis, were deemed the likely reservoirs of *qnrVF*. As of March 2022, there were exactly six sequences deposited in NCBI containing *qnrVF1* (V. furnissii 104486766 and V. furnissii VFN3 carried plasmid-borne *qnrVF1*; three Morganella morganii strains and Providencia huaxiensis WCHPr000369 harbored chromosomal *qnrVF1*). It is tempting to speculate that *qnrVF1* emerged recently and that this gene has not yet spread globally. Further monitoring of *qnrVF1* in clinical and aquatic environments is needed. We also found that, similar to QnrVC, QnrA, QnrB, QnrC, QnrD, QnrE, and QnrS (1, 13), QnrVF1 plays important roles in low-level resistance to ciprofloxacin and nalidixic acid (Table S2). Further structural and biochemical studies of QnrVF1 may shed light on the mechanism by which QnrVF1 mediates quinolone resistance. Since quinolones are widely used drugs (1), the emergence of *qnrVF1* may jeopardize the battle between antimicrobials and bacteria. In addition, *qnrVF1* genes are all flanked by IS*CR1* (Fig. 1C), indicating that IS*CR1* may have an important role in the mobilization of *qnrVF1*. This is similar to other *qnr* family genes: IS*CR1* is also involved in the mobilization of *qnrVC4*, *qnrA1*, *qnrA6*, *qnrB2*, *qnrB4*, *qnrB6*, and *qnrB10* (13). Although both p104486766-qnrVF1 and pVFN3-blaOXA-193K possess *qnrVF1* and belong to the same group of replicon type plasmids, only pVFN3-blaOXA-193K was found to be transferable (14). This suggests that pVFN3-blaOXA-193K could mediate the spread of *qnrVF1*.

In conclusion, we identified a novel *qnr* family gene, *qnrVF*. To the best of our knowledge, p104486766-qnrVF1 is the first reported plasmid that carries *qnrVF1* in *Vibrio* species. We also found that *qnrVF1* is involved in quinolone resistance. IS*CR1* may facilitate the spread of *qnrVF1*. The spread of *qnrVF* may pose a considerable threat to public health.

### Data availability.

The complete sequences of strain 104486766 were deposited into GenBank with accession numbers CP100422 to CP100424.
