# Supplementary material for: Identification of qnrVF in a Multidrug-Resistant Vibrio furnissii Clinical Strain
Source: Microbiol Spectr. 2023 Jan 19;11(1):e01934-22. doi: 10.1128/spectrum.01934-22 (PMC9927268; doi:10.1128/spectrum.01934-22)
Supplement: Supplemental file 1 — Tables S1 and S2 and Fig. S1. Download spectrum.01934-22-s0001.pdf, PDF file, 0.2 MB [file spectrum.01934-22-s0001.pdf]

Table S1 Antimicrobial susceptibility testing results (MIC, mg/L) of *V. furnissii* 104486766. Three independent experiments were performed.

Strain	CFF	CRO	CAZ	AZM	AMP	ATM	MER	IMP	CIP	TET	STR	CHL	COL	TIG
V. furnissii 104486766	8/R	8/R	16/R	64/R	>150/R	2/S	0.25/S	2/I	32/R	32/R	8/S	125/R	0.25/S	0.25/S
E. coli ATCC 25922	0.25	0.25	0.25	0.5	1	0.5	0.1	0.1	0.25	0.25	0.5	0.5	0.1	0.1

CFF, ceftiofur; CRO, Ceftriaxone; CAZ, ceftazidime; AZM, Azithromycin; AMP, ampicillin; ATM, aztreonam; MER, meropenem; IMP, imipenem; TET, tetracycline; TIG, tigecycline; STR, streptomycin; CHL, chloramphenicol; COL, colistin; CIP, ciprofloxacin. *E. coli* ATCC 25922 was used as quality control. S, susceptible; I, intermediate; R, resistant.

Table S2 Antimicrobial susceptibility testing results (MIC, mg/L) of *E. coli* transformants to quinolones. Three independent experiments were performed.

Strain	Ciprofloxacin	Nalidixic acid
BL21(DE3)/pET28a-*qnrVF1*	0.05	8
BL21(DE3)/pET28a	0.0125	1

Fig. S1 Circular comparisons of p104486766-qnrVF1 and related plasmids, pKP-16-57-NDM-1, pEc-13-49-NDM-1, pVFN3-blaOXA-193K, pPV835TEM24, and pVC1447. The outermost circle denotes p104486766-qnrVF1 with arrows for coding genes.

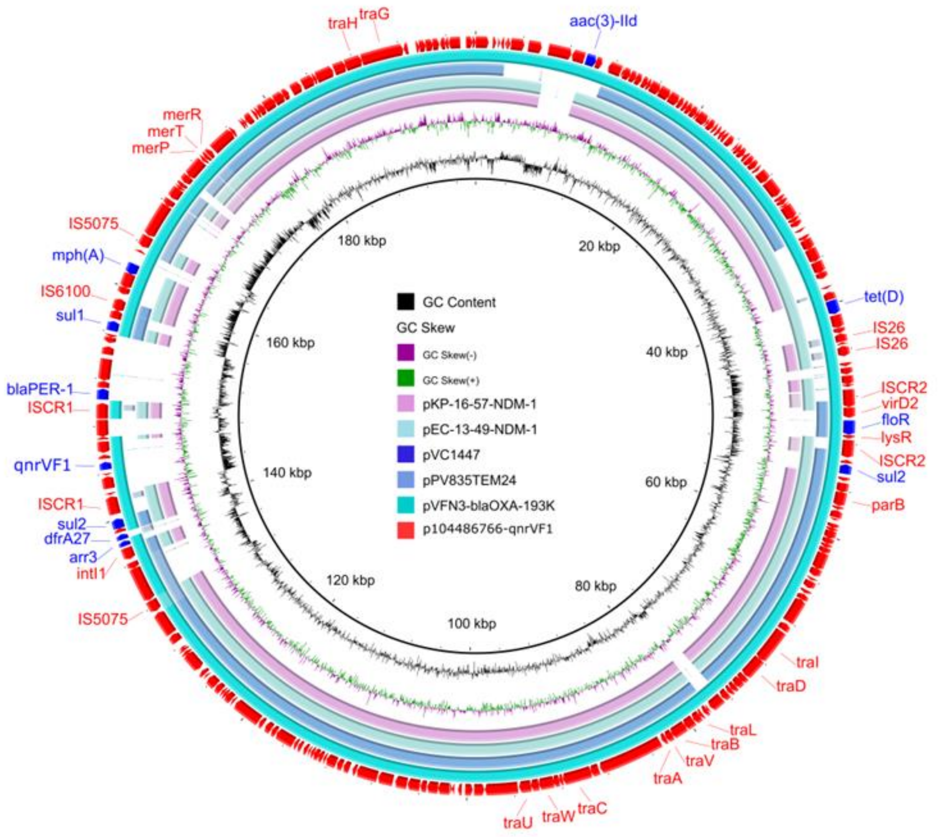

traH
traG
aac(3)-IId
merR
merT
merP
IS5075
mph(A)
IS6100
sul1
blaPER-1
ISCR1
qnrVF1
ISCR1
sul2
dfrA27
arr3
intI1
IS5075
tet(D)
IS26
IS26
ISCR2
virD2
floR
lysR
ISCR2
sul2
parB
traI
traD
traL
traB
traV
traA
traC
traW
traU
180 kbp
20 kbp
160 kbp
40 kbp
140 kbp
60 kbp
120 kbp
80 kbp
100 kbp
GC Content
GC Skew
GC Skew(-)
GC Skew(+)
pKP-16-57-NDM-1
pEC-13-49-NDM-1
pVC1447
pPV835TEM24
pVFN3-blaOXA-193K
p104486766-qnrVF1
